# "Plastic Filling" as a "Power" in Dentistry

**Published:** 1878-12

**Authors:** J. Foster Flagg


### ARTICLE II.

### "*Plastic Filling*" *as a* "*Power*" *in Dentistry.*

BY J. FOSTER FLAGG, D. D. S.

Read before the American Dental Association, August, 1878.

MR PRESIDENT AND GENTLEMEN:

As the result of more than thirty years' experience in the practice of dentistry, I find myself in the front ranks as an ardent, earnest, conscientious advocate for the use of "plastic" materials for filling teeth.

As the chosen ennunciator of the "basal principles of the

New Departure," I spoke on behalf of my friends, Drs. Palmer and Chase, at the last November meeting of the New York Odontological Society.

I spoke then for "basal principles," which should so govern practice as that a great advance might be made in the *saving of teeth* over that, which you all know has resulted from the practice of the past.

I recognize as well as you do the high appreciation which the whole world extends to American dentistry.

I recognize as well as you do the wonderful manipulative skill of our "best operators."

I recognize as well as you do the vast amount of good which has been done to the community and the world by all this hearty effort, but I also recognize that *in despite of it* our people are *semi-edentulous*, and that the whole civilized world is flooded with *artificial dentures.* Dentures on celluloid, dentures on rubber, dentures on silver, and dentures on gold, dentures on porcelain, and dentures on platinum, dentures on everything except the roots which God designed for their support.

It is too much for me to claim that this result is due largely to the teaching that *gold* is the *best* material with which to *save* teeth ?

Gentlemen, my plans for life (if life is spared) are such as that it is most probable that many years will pass before I meet with you again, if ever, and I therefore desire the more to leave with you that which I have brought, most solidly, most clearly, and with the utmost kindness.

To those of you who differ with me most entirely, I ask that you will *permit* an honest difference of opinion ; that you will accord to me sincerity of purpose, and that while you combat *my views* to the utmost, you will not expend any strength in *personal* antagonism.

*I* am nothing in this ; I merely advocate principles and practices which must stand or fall solely upon their own merits.

If I can say to you that the practice which I advocate is

*eminently* successful, and you have no *reasonable cause* for discrediting my assertion, it is your *duty* to your patients and your profession that you examine well its workings, and make yourself so conversant with its minutiæ as to be *fitted* to express opinions, rather than be content to pass it by in wholesale condemnation.

If I can say to you that with the gradual abandonment of gold I have *also* equally come to the abandonment of artificial dentures, it behooves you to look to it and *see* the connection which exists, if there be any, between the two.

Is gold the easiest material with which to fill? Is it not the most difficult?

Is gold the quickest material with which to fill? It is not the greatest consumer of time?

Is gold the most comfortable material with which to fill? Is its use not usually attended with more discomfort than any other filling?

Is gold the most economical material with which to fill? Is it not, by far, the most expensive?

Is gold the most durable material with which to fill?

I ask you, gentlemen, is it *possible* that your experience is so adverse to mine as that you can say that in the teeth which *most need* saving, gold is the most *durable* of all your fillings?

Then, if gold is the most *difficult*, the most *tedious*, the most *uncomfortable*, the most *expensive*, and *not the most durable* material with which to fill teeth, is it not a *reasonable* deduction that its almost exclusive use, and its constant adulation, its upholding as the *test* for dental ability, and its universal acceptation as the *best* of filling materials, has *much* to do with all this flood of pulpless teeth, of alveolar abscesses, discolored crowns and jagged roots, of toothless jaws and plates of india-rubber?

I put this *strong*, gentlemen, that you may see how it looks to, at least, one of you; that you may be led to think that *if* it *can* so look to anyone, perhaps it may be worth your while to make examination of that which seems *at first* absurd.

*I tell you* my patients do not think that it is *absurd.*

I assure you those that have had decades of years of past experience are loud mouthed in confirmation of what I say to you.

Hundreds of patients have been for years' comparing notes with the results in mouths of friends, and their testimony corroborates my own experience.

Hundreds of patients who have tried for years "accepted dentistry," regardless of time, suffering, and expense, and who now for years have tried the dentistry which I advocate before you, have not words sufficiently expressive of their condemnation of the former, or of their satisfaction with the latter.

It is not I alone who can tell you this; my office has been no "closed *sanctum.*" Its door has ever been open, and many, I am happy to say, have brightened my rooms with the light of their faces.

They have both *seen* and *heard* this that I claim for our "New Departure" practice. I have plenty of words and letters which assure me of the great modifications in practice which have grown out of these visits.

If all of you do not know me well enough to feel assured that I tell you of these things *because* I feel that I should be derelict in my duty to my profession and to you were I not to do it, I am happy in the conviction that some, *and indeed many* of you, do me the justice to believe this.

I desire that you shall know of these *possibilities.* I desire to have no *secrets.* I desire to have the privilege of saying to you that I practice from an entirely different stand-point from that which, up to this time, has been given by text-books and colleges.

I desire to tell you of the *comfort* which comes from this practice, both to patient and operator.

I desire to tell you of the *ease* with which apparently insurmountable dental difficulties are overcome.

I desire to tell you of the *satisfaction* with which each year's successes are added to those of all the previous years.

I desire to tell you of the feeling of proper pride—aye! *thankfulness*—with which I see the years roll on, while arches by the hundreds remain unbroken.

I desire to tell you how *economically* all this is done; how promptly and willingly bills are paid; and how frequently words of good cheer and commendation accompany "checks" for services rendered.

But, gentlemen, I desire, above all, to say to you that I attribute all this mainly to the use of "plastic filling."

I wish to say to you, at this time, that with the practice which some of you *know* I have, and with the character of practice which you may, all of you, *infer* I have, and in a city which has just claim to rank high as proficient in dentistry, I have not used one sheet of gold foil for almost two years, and have sailed for the past seven months with "no gold used" in capital letters upon my appointment cards.

I wish to say this *openly*, for the comfort and support of those who desire to *use* plastic filling materials.

Gentlemen say that our promulgation of such practice will lead to wholesale, unworthy use of plastic filling.

That is no affair either of ours or theirs. Let them learn to use them *worthily*, and thus *by example*, rather than precept, show our erring brothers the error of their ways.

But while I advocate thus strongly the use of *plastic filling materials*, and while I state to you that I have completely abandoned gold, I wish you distinctly to understand that this practice is not now, *and never has been*, advocated by the "New Departure."

In the presentation of our desires, it was expressly stated just what kinds of teeth should be filled with gold, and just what kinds of teeth should be filled with plastic fillings.

This having been misstated and misrepresented, the assertion is now made that what we desire is no "New Departure," but is just what has long been practiced as "eclectic dentistry."

Now this is just as far from truth as were the first misstatements.

That plastic filling materials have been used in a sort of clandestine manner, and as desperate "last resorts," we admit.

That their employment was generally regarded as *derogatory* to "first-class" ideas, and that the fillings were stamped as "second-class," we contend.

And we insist that while even *excellent* practitioners admitted that they occasionally employed gutta percha, oxychlorides of zinc, and even amalgam, there were still some who asserted that they "never used anything but gold," and who from this claimed a *superiority*, which by the members of the profession at large was tacitly accorded them.

In contradistinction to all this,—in marked deviation from all this,—we desire that a "New departure" practice shall recognize that, for certain *definite* kinds of teeth, and for cavities in certain *definite* places, for which at present gold is regarded as, and stated to be, "the best," other materials than gold are *better* than gold; that they will preserve teeth better, and that *therefore* gold is *not entitled* to the broad credit of being the "best" material for filling teeth.

We wish it to be understood by the profession, and secondarily by the community, that, even if gold is the "best" material for the *hard*, *strong teeth* and the easy places, it has to take a *subordinate* position when the *soft* teeth and the *dreadfully difficult* and inaccessible places are marched to the front, for that *gold* is not then our best weapon for defense.

And more than this, we insist upon it that just in proportion as teeth are more and more in need of a *preserver*, so does gold *less* and *less* meet the requirements, and this *too* even in the hands of the *skilled few* who cannot care for the one-thousandth part of the teeth that need saving.

Our "basal principles," we think, must become "accepted." Gold must be known and recognized as not the best in many, many cases.

The mere fact of "leakage" must be *known* to be not *necessarily* detrimental.

It must be *known* and acknowledged, that while "leakage" is *fatal* to the integrity of a good filling, it is not practically detrimental to one of gutta-percha, while again it, in some instances, is the very thing which effectually arrests decay under fillings of tin and of amalgam, thus causing them to preserve, for a long lifetime the teeth which are so soon lost in ordinary practice with the use of gold.

Compatibility of filling material with tooth-bone must be *known* to be the great foundation-stone for the successful saving of the teeth, and such splendid materials as gutta-percha, amalgam and the zinc plastics must *not* be taught to the *rising generation* of operators as "temporary," "vile," "worthless," and "ruinous to teeth," and their employment as "tending to *degrade dentistry!*"

And now, gentlemen, I wish to call to your notice what a "power" this plastic filling has become in dentistry.

It is *even now* a very well worked-up *specialty* of our profession; one that is so little known as scarcely to be mentioned except with contempt or disapprobation from the *lecture-stand*, and yet, one which can now take any denture so forlorn as to be hopelessly abandoned by the best gold operator in the world, and make of it a comfortable, satisfactory and beautiful success.

This is a marvelous claim, but those of you who best know my record will not for a moment think that I would say anything which I could not substantiate.

I have been doing this for years; it is now nearly ten years since I passed the "half-way" station at which I filled fifty *per cent.* of all my cavities with "plastic fillings;" as I have told you, it is now almost two years since I have u ed any gold, and during all this time the "hardest" kind of practice, in the shape of the "softest" kinds of teeth, has been aggregating apace.

I have, to the full, those "very soft teeth which" (as a member of the New York Odontological Society lately said) "we all dislike to see at the very first sitting," and there is no such feeling engendered by their presence. It is only a source of satisfaction, as I inform the patients of the ease and comfort which I can give them in place of the pain, discomfort, and dissatisfaction which they have before experienced.

It is only a source of increased incentive, as year after year demonstrates to them, as well as to me, the strength of my position; and the constant and frequent recurrence of such remarks as, "How different this is from what it used to be!" "How long it is since I have lost a tooth!" "How glad I am I learned of this!" and such outcroppings of gratitude and satisfaction, lighten my labors, make me thankful for the instrumentalities which enable me to bestow such blessings, and cause me to wonder if it *can be* that it will be *long yet* before hundreds will be *educated* to leap joyfully over the long road which I have so slowly traveled.

And such a practice as this I tell you of is spoken lightly of—yes, worse than lightly—by the last guard of "accepted dentistry,"—men who know nothing of our work, who know nothing of our operations, who know nothing of our tooth-saving, beauty making, comfort-giving, permanent combinations of our plastic fillings; men who know nothing of the proportions, or even of the components of our materials, who do not even know their names! much less their qualities or modes of working; who would not know their proper uses or their proper using if they were given them; and yet these men condemn it all, and in no measured terms, and thus *teach dentistry!*

Until quite recently (!) scarcely any one has claimed to use amalgam *much*, and yet, for many years, the steady increase of demand has been supplied by Townsend's, Walker's, Ash's, Lawrence's, Smale's, Arrington's, Moffitt's, Ashmead's, Hood & Reynold's, Holmes's, Diamond, Luther's, Sullivan's, Fletcher's, Welch's, Davis's, Dunlevy's, Chicago (Gold and Platina,) Weston's (S. S. White's,) Standard (Eckfeldt & Du Bois's,) "Best" (Spencer & Crocker's,) Stannous Gold ("O. P." Chase's,) Sterling (Davis & Leyden's,) Extra (Johnson & Lund's,) and Hardman's of Muscatine, Iowa; and these alloys, so far from being "*all the same*," are composed, some of two metals, some of three metals, some of four metals and some of five metals; each has certain individual characteristics; each has its rapidity or its slowness of "setting;" each has its grade of shrinkage, though some say "no shrinkage;" each has its grade of edge strength, its liability to discoloration, its extent of bulging, and its value as a filling material for saving teeth. All this should be recognized by the professors of operative dentistry, and all the facts in regard to each should be taught as a part of dental education; for amalgam will save, for it has saved, many a tooth that had been tried by the best operators unsuccessfully with gold.

For gutta-perchas we have, for red, selected "base plate," and for white, we have Hill's, Bevan's, Jacob's, Codman's, Johnston's, White's, George's, Johnson & Lund's, Caulk's, and many others of lesser note; each of these has its rank, its heat test, its appropriate place, its proper method of manipulation, and, as these are known, its value.

For oxychlorides we have Sorel's, as the first, and type of all, followed by Metcalf's, Pearson's, Houghton's, Roberts's, Kellnitz's, Smith's, Fletcher's, Poulson's. Worff's, Cement Plombe, Rock Cement, Guttensohn, Franzelius's, Crystalline, German Cement, Agate Cement, Acme Cement, Ludwig's, and Foundation.

Then we have the oxysulphates, which are mixed with mucilage or clear water, are non-irritating, and used for protecting pulps; and finally, the oxyphosphates, of which we have already some eight varieties, the most notable of which are Poulson's (six shades,) Grass & Worff's "Zinc Pyrophosphate," and Jiletti's "Piro-osseina" and "Pirosmaltina."

Is there not a host of them? and do you suppose that gentlemen who have but infrequently used any of them know all about all of them? I think not!

Every kind of "plastic filling material" has its own decided peculiarities of manipulation, during which it is found necessary to employ for explanation and comparison of views such terms as "weighing, rubbing, mixing, washing, heating, setting, tapping, trimming, bulging, talcking, cold-soldering, capping, guarding, softening, lining, pelleting, whitening, wafering, buffering, domeing, facing," and the like,—terms, each of which conveys to the mind of the plastic-filler the idea of some definite process, action of material, manipulative result or specific means for the accomplishment of some desired end.

So far from all this being "*guess work*," we have a range of "tests" which tell us very well what we may expect of any material under given conditions. These are such as strength tests, edge test, setting test, shrinkage test, expansion test, color test, heat tests (wet and dry,) leakage test, froting test (for probable wear,) acid test, alkali test, conduction test (electrical and thermal,) and, finally, the oral test, which decides the compatibility of materials with tooth-bone and their behavior in the oral fluids and under oral influences.

By means of these tests we are enabled to make a choice of material to meet the varied indications that constantly present in practice, which, to our apprehension, approaches to something like science.

It is by these means that we frequently combine two, three, four, or more different materials in the filling of one cavity, each of which best subserves its purpose in its appropriate position, and insures an operation which for comfort, beauty, and permanency can in no other way be equaled.

It is a source of no little satisfaction to me that I can say that my office has been an open "clinic room" for many years; students of a week, graduates of a year, and venerable practitioners of almost half a century's experience, have been alike welcome, and have seemed alike satisfied. They have each left their contribution to my store for dissemination, and they have each told me that they took away, in return, something which compensated them for their visit.

It has always been a pleasure to me to answer notes of inquiry, and when in my power, to give the desired information.

My relations with the class, as incumbent of the chair of Dental Pathology, were of an exceedingly pleasant nature, warming frequently into close personal friendship. I have enjoyed an intimacy with men in our profession which has made me feel that it *must* of necessity include some of the choicest distillations of humanity; and it is, therefore, with no ordinary feelings that I have come to this meeting, bringing with me the conviction that it must be "for years, and it may be forever," that our *associate relations* will be severed. I feel that, *professionally*, my *associate life-work* has been done. I feel that it has been vouchsafed to me to open before you a professional pathway which is one of success, relief, and compensation to *you*, and of great comfort and satisfaction to your *patients*. I feel that in its pursuance you can, as I have done, practically ignore mechanical dentistry,—that boon, that almost priceless blessing, to those who require it; but how much better *not to require it!* With the same energy that has been expended upon "gold" directed in the channel of "plastic filling," the children of our children should view an artificial denture as a curiosity. My patients have no knowledge of them. Why should yours have?

But to accomplish this you have a big field to plow; it has to be harrowed, planted, and cultivated; by these means *only* can you secure a crop. But the harvest is worthy all the labor you can give. It makes of dentistry a *blessing* to the patients, and a *luxury* to its practitioners. I *know*, gentlemen, *because I have it so!* I have faith to believe that it will be given to you, and that you will find it so, and in that faith *I leave it with you.—Cosmos.*

